# Characterization of Green Fluorescent Protein in Heart Valves of a Transgenic Swine Model for Partial Heart Transplant Research

**DOI:** 10.3390/jcdd10060254

**Published:** 2023-06-10

**Authors:** Katherine Bishara, Jennie H. Kwon, Morgan A. Hill, Kristi L. Helke, Russell A. Norris, Kristin Whitworth, Randall S. Prather, Taufiek Konrad Rajab

**Affiliations:** 1 Department of Surgery, Medical University of South Carolina, Charleston, SC 29501, USA; 2 Department of Comparative Medicine, Medical University of South Carolina, Charleston, SC 29501, USA; 3 Department of Regenerative Medicine, Medical University of South Carolina, Charleston, SC 29501, USA; 4 National Swine Resource and Research Center, University of Missouri, Columbia, MO 65211, USA; whitworthk@missouri.edu (K.W.);

**Keywords:** transgenic, heart transplant, immunohistochemistry, swine model

## Abstract

A transgenic strain of pigs was created to express green fluorescent protein (GFP) ubiquitously using a pCAGG promoter. Here, we characterize GFP expression in the semilunar valves and great arteries of GFP-transgenic (GFP-Tg) pigs. Immunofluorescence was performed to visualize and quantify GFP expression and colocalization with nuclear staining. GFP expression was confirmed in both the semilunar valves and great arteries of GFP-Tg pigs compared to wild-type tissues (aorta, *p* = 0.0002; pulmonary artery, *p* = 0.0005; aortic valve; and pulmonic valve, *p* < 0.0001). The quantification of GFP expression in cardiac tissue allows this strain of GFP-Tg pigs to be used for future research in partial heart transplantation.

## 1. Introduction

Green fluorescent protein-transgenic (GFP-Tg) animal models are often used in research as a direct model for the visualization of cellular markers [1]. Recent advances in genetic medicine have allowed easier transfection of transgenes in larger animal models [2]. Pigs are the preferred non-primate large animal model for translational research due to their wide availability, short period to reproductive maturity, rapid growth, low maintenance cost, and similar anatomy and physiology to humans [3,4]. This is especially true for cardiac translational and surgical research as pigs’ cardiac anatomy and physiology closely match that of humans with few differences [4].

GFP-transgenic pig strains expressing GFP ubiquitously as well as in specific tissues have been created and are currently being used in translational research [5,6,7]. Both Kawarasaki, Uchiyama [8] and Brunetti, Perota [9] describe strains of transgenic pigs that ubiquitously express GFP using a cytomegalovirus and chicken beta-actin (CAG) promoter, created with somatic cell nuclear transfer (SCNT). Both groups have described the expression of GFP in multiple tissues, including cardiac tissues. Brunetti et al. [9] confirmed GFP expression in myocardium and endocardium tissues whereas Kawarasaki et al. [8] confirmed this only in the myocardium. Neither group has described GFP expression in cardiac valves or great arteries, specifically.

Recently, a new strain of GFP-Tg pigs was described by Whitworth et al. [10] which uses a chicken β-actin/rabbit β-globin hybrid promoter (pCAGG) to drive enhanced-GFP (EGFP) expression. Their characterization included confirming the expression of EGFP in blastocyst-stage embryos after somatic cell nuclear transfer, in the spherical conceptus at day 12, in the eye of a day 30 fetus, and macroscopically in a 9-day-old piglet. However, the characterization and quantification of EGFP expression in cardiac tissues have yet to be performed in this strain. This strain of GFP-Tg pigs was performed using methods that resulted in 100% pregnancy rates and ubiquitously expressed GFP whereas other strains using similar transfection methods resulted in mosaicism.

In this paper, we aim to characterize GFP expression in the semilunar valves and great arteries of this GFP-Tg strain. As pigs are the large animal model of choice for cardiac surgery research and because ubiquitous GFP expression can trace cellular origin in transplantation studies, this analysis can potentiate research in heart transplantation as well as valve replacement by using allograft or xenograft valvular tissue. Additionally, this strain is a farm pig making it ideal for cardiac surgery research due to the similar growth rate and size to humans. Here, we characterize the GFP expression in the semilunar valves and great arteries of GFP-Tg pigs compared to wild-type. Immunofluorescence staining was performed to visualize and quantify GFP expression in the aortic valve, pulmonary valve, aorta, and pulmonary artery of GFP-Tg pigs.

## 2. Materials and Methods

**Figure 1:**
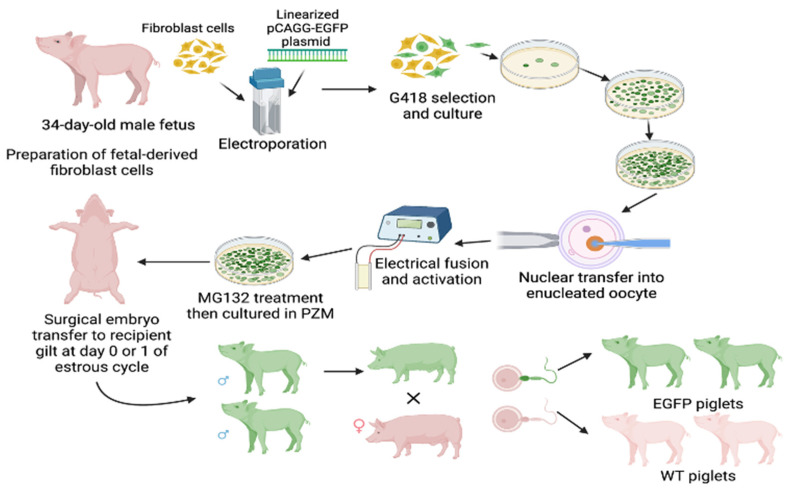
Schematic of transfection of pigs using pCAGG-EGFP plasmid, electrical fusion/activation, and propagation of transgenic strain. Created with BioRender.com (accessed 28 March 2022).

The development of GFP-transgenic swine using pCAGG promoter and fusion/activation is detailed in Whitworth et al. (Figure 1) [10].

### 2.1. Propagation of GFP-Transgenic Pigs

Sexually mature large white crossbred gilts were artificially inseminated with semen collected from a heterozygous EGFP (RRID NSRRC:0016 GFP NT92) mature boar on day 0 or 1 of estrus detection (Figure 1). Pregnancy was confirmed by estrus detection and subsequent ultrasonography. The pregnancy progressed to approximately 114 days post-estrus. The pigs were farrowed naturally, and EGFP expression was determined in the piglets by visual examination with BLS-LTD UV goggles (BLS, Budapest, Hungary). The piglets were ABO-typed by using an Eldoncard INC rapid blood type test. One wild-type and one EGFP-positive piglet with matching O or B blood types were selected for the experiments. Piglets (GFP-Tg, n = 3; WT, n = 3) were weaned at 3 weeks and sacrificed at 6 weeks of age, and their hearts were harvested for analysis.

### 2.2. Macroscopic GFP Visualization

Piglets were visualized at 30 days of age by using the Xite Fluorescence Flashlight System (Xite-RB-GO, Excitation 440–460 nm, Barrier filter glasses 500–560 nm bandpass; purchased from Electron Microscopy Sciences (Hatfield, PA, USA)).

### 2.3. Tissue Processing

#### 2.3.1. Dissection

The aorta and pulmonary arteries were individually dissected free, and transverse sections of the great arteries were harvested. The aortic and pulmonary valves were harvested as individual sinuses of Valsalva by separating each leaflet at the arterial wall between leaflet commissures.

#### 2.3.2. Tissue Fixation and Processing

Tissues were placed in 10% formaldehyde for 12–18 h and then placed in PBS at 4 degrees Celsius. Tissues were dehydrated in sequential ethanol steps and placed into Histo-Clear (Electron Microscopy Solutions (Hatfield, PA, USA), 64111-04) overnight. On the second day, the tissues were paraffinized and embedded. Sectioning was performed at 4 μm on polarized slides.

### 2.4. Immunofluorescence

Slides were deparaffinized in xylene and rehydrated with decreasing concentrations of ethanol. Antigen retrieval was performed by using IHC-Tek™ Epitope Retrieval Solution (IHC World (Ellicott City, MD, USA), IW-1100-1L) in a steamer. Autofluorescence was blocked by using Image-iT FX Signal Enhancer (Invitrogen, Thermo Fisher Scientific, (Waltham, MA, USA), cat. no. I36933). The protein was blocked by using Normal Goat Serum, 2.5% (Vector Laboratories (Newark, CA, USA), S-1012), and then incubated overnight with primary antibody (rabbit anti-GFP IgG 1:1000 (Abcam (Cambridge, MA, USA), ab5665)) diluted in Antibody Diluent (Dako (Santa Clara, CA, USA), S0809). Slides were incubated the next day in diluent with goat anti-Rabbit IgG (H+L) Highly Cross-Adsorbed Secondary Antibody, Alexa Fluor™ Plus 555 (Invitrogen, A32732) at 1:100. Autofluorescence was quenched by using Vector TrueVIEW (Vector Laboratories, cat. no. SP-8400), and nuclei were counterstained with antifade mounting medium with DAPI Hardset (Vector Laboratories, H-1200).

### 2.5. Microscopy

Images for both immunofluorescence (IF) slides were taken with a Keyence All-In-One Fluorescence Microscope (BZ-X810, Keyence Corporation (Itasca, IL, USA)). GFP staining was visualized by using the BZ-X Filter TRITC (OP-87764, Keyence) and DAPI was visualized with BZ-X Filter DAPI (OP-87762, Keyence). Images for measurement were taken at 20× magnification. Exposure times were 1/15 s for DAPI and TRITC (GFP). Each section on a single slide was imaged to provide technical replicates. Images were analyzed by using BZ-X software. The colocalization of GFP and DAPI was measured at a threshold of 60 red and 0 blue. DAPI alone was measured with a threshold of 7. IF images for publication were taken with Keyence by using Z-stacks, and the TRITC filter used to visualize Alexa Fluor™ Plus 555 was changed to green to represent GFP.

### 2.6. Statistics

The area (μm^2^) of colocalization of GFP with DAPI of each image was divided by the area of DAPI to account for the total amount of tissue captured in each image. Statistical analyses of the microscopy data were performed by using GraphPad Prism (version 9.3.1 for Mac OS, GraphPad Software, San Diego, CA, USA, www.graphpad.com, accessed on 22 May 2022). Mann–Whitney tests were used to analyze the colocalization/DAPI data. A *p*-value < 0.05 was considered statistically significant.

## 3. Results

### 3.1. Propagation of GFP-Transgenic Pigs and Visualization of GFP Macroscopically

Macroscopically, the GFP-positive pigs had slightly green-tinted hooves without the use of the Xite flashlight. With the flashlight, the hooves, snout, and mucus membranes of the GFP-positive pig fluoresced green compared to the WT pig. Perioperatively, the cardiac tissue had a green tint.

### 3.2. Microscopy of Immunofluorescence of GFP Tissues

**Figure 2:**
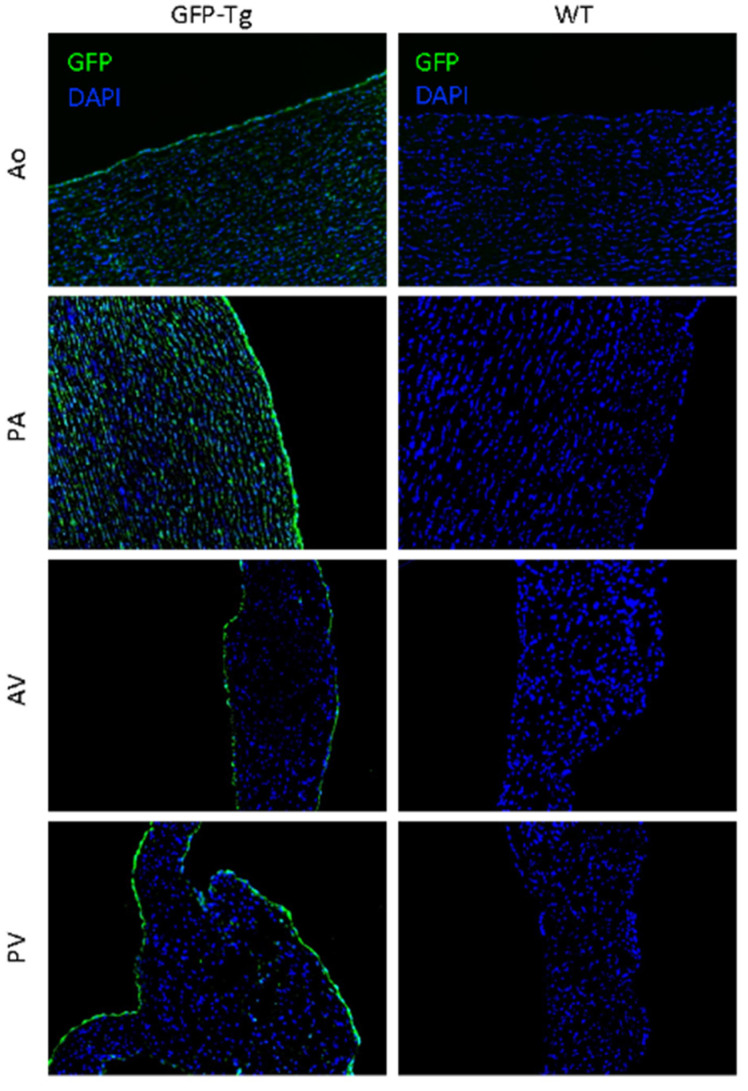
Immunofluorescence images of the aorta (Ao), pulmonary artery (PA), aortic valve (AV), and pulmonary valve (PV) of green fluorescent protein-transgenic (GFP-Tg) and wild-type (WT) pig tissues stained for GFP (green) and nuclear staining (DAPI; blue). Scale bar = 100 μm.

Visualization of GFP by immunofluorescence showed GFP in all selected cardiac tissue of the GFP-Tg pigs (Figure 2). The endothelium of the great arteries and semilunar valves had more concentrated GFP expression due to the greater concentration of the endothelial cells compared to the tunica intima or tunica media of the great arteries and interstitium of the valves.

**Figure 3:**
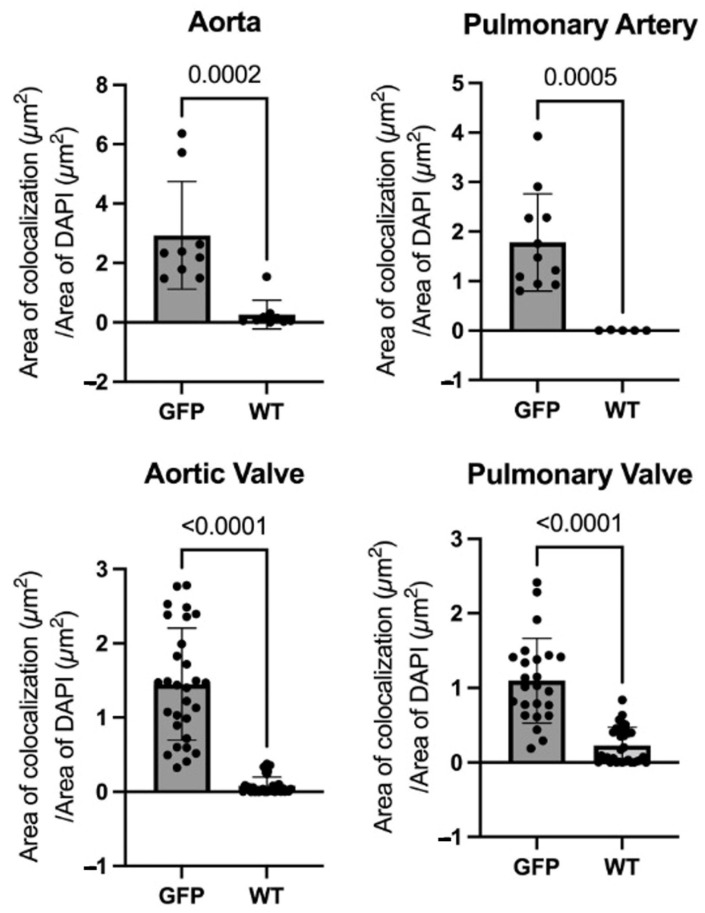
Areas of colocalization over areas of DAPI were measured for green fluorescent protein (GFP) vs. wild-type (WT) in the aorta (Ao), pulmonary artery (PA), aortic valve (AV), and pulmonary valve (PV). GFP-transgenic (GFP-Tg) cardiac tissue expressed GFP at a statistically significantly higher level than WT cardiac tissue in all four tissues (Ao, *p* = 0.0002; PA, *p* = 0.0005; AV and PV, *p* < 0.0001). Mann–Whitney statistical test.

All four types of cardiac tissues measured had statistically significantly higher GFP expression in the GFP-Tg tissue compared to the WT tissue (Ao, *p* = 0.0002; PA, *p* = 0.0005; AV and PV, *p* < 0.0001) (Figure 3).

## 4. Discussion

The expression of GFP was visualized and quantified in GFP-Tg swine cardiac tissue compared to wild-type cardiac tissue in the aortic valve, pulmonic valve, aorta, and pulmonary artery. In each type of cardiac tissue, GFP expression was greater in the GFP-Tg tissue versus wild-type, with wild-type tissue having minimal fluorescence. Therefore, GFP-Tg valvular tissues in this strain of GFP-Tg pigs have sufficient levels of GFP expression to be used in future translational and surgical research to compare to wild-type tissues.

We investigated the GFP expression of this strain of transgenic pigs due to its ubiquitous GFP expression and propagation that allowed for the comparison of GFP-positive offspring to its wild-type sibling [10]. Additionally, as no cardiac research has been performed with this strain, we would be able to establish a baseline of GFP expression in cardiac tissue, as performed here, so that we may utilize our findings for future surgical experiments.

In living animals, green fluorescent protein, as a genetically encoded fluorescent marker, offers reliable and efficient means to observe the destiny of cells [1]. In transplant research, it can be used to easily visualize the integration or rejection of recipient/donor cells. To this point, GFP-transgenic pigs have not yet been used for research in solid organ transplantation. GFP-transgenic pigs have been used for embryonal retinal cell transplants to wild-type adult pigs [5,11] and arthroscopic knee cartilage repair [12]. GFP models using smaller animals have been used to demonstrate transplantation such as fetal liver transplantation in mice [13] and corneal transplant in rats [14]. All the above studies were able to phenotypically demonstrate the integration and/or differentiation of transgenic cells after transplantation. GFP allows for the identification of cell origin using simple immunofluorescence rather than using multiple genotype-specific markers. The characterization of GFP expression in cardiac valve and great artery tissue provides the necessary baseline for this strain of transgenic swine to be used in non-transplant cardiac research, pig-to-pig cardiac allotransplant models, and pig-to-non-human primate cardiac xenotransplant or xenograft, similar to research performed in non-pig animal models and non-cardiac pig tissues as described above.

Future directions of this work include the use of the GFP-Tg tissue in partial heart transplantation. This entails using fresh valve tissue from a donor heart to replace the corresponding valve in the recipient [15,16]. This procedure was proposed to be an alternative for pediatric patients with unrepairable valve defects as the current options are limited. Mechanical, homograft from cadavers, and xenografts each have individual pitfalls, but all have limited size options and do not grow with the patient resulting in high reoperation rates leading to morbidity and mortality [15]. The mortality of pediatric patients waiting for a heart transplant is high [17,18], and the probability of rejection or graft dysfunction increases with time [19]. Valve tissue appears to be immune-privileged to an extent, suggesting that decreased immunosuppression would be required [20]. This concept has been demonstrated by using cadavers to determine surgical feasibility, and the logistical feasibility is currently being investigated [21]. Additionally, the demonstration of this procedure is being performed in piglets. The GFP-piglet’s pulmonic valve is transplanted into the wild-type piglet recipient and will be used to assess the viability of donor cells and the endothelialization of the donor valve by recipient cells. The analysis of GFP expression in the GFP-Tg cardiac tissue, specifically the valves, compared to wild-type presented here will provide the baseline data necessary for comparison to GFP expression in potentially endothelialized GFP-positive donor valves in wild-type recipient pigs.

The limitations of this study include a lack of staining for collagen, decreased visual detectability of GFP fluorescence in the interstitium of the valve tissues, and uneven numbers of samples that were able to be visualized. As seen in Figure 2, several WT images had green fluorescence expression due to background and overexposure as all images were taken at equal exposures and slides were stained in different batches.

## 5. Conclusions

A GFP-transgenic strain of pigs expresses GFP at a statistically significantly higher level than wild-type cardiac tissue in the semilunar valves and great arteries. Confirmation of this expression allows this strain to be used in cardiac research where GFP can be used as a cell marker. Current research involving partial heart transplantation will be able to use this characterization of GFP as a baseline comparison for transplanted valves from GFP-transgenic donors to wild-type recipients.

## Data Availability

The data presented in this study are openly available in FigShare at 10.6084/m9.figshare.23426372.
